# Aggressive local treatment for recurrent intrahepatic cholangiocarcinoma—Stereotactic radiofrequency ablation as a valuable addition to hepatic resection

**DOI:** 10.1371/journal.pone.0261136

**Published:** 2022-01-04

**Authors:** Eva Braunwarth, Peter Schullian, Moritz Kummann, Simon Reider, Daniel Putzer, Florian Primavesi, Stefan Stättner, Dietmar Öfner, Reto Bale

**Affiliations:** 1 Department of Visceral, Transplant and Thoracic Surgery, Medical University of Innsbruck, Innsbruck, Austria; 2 Department of Radiology, Interventional Oncology—Microinvasive Therapy, Medical University of Innsbruck, Innsbruck, Austria; 3 Department of Internal Medicine I, Gastroenterology, Hepatology & Endocrinology, Medical University of Innsbruck, Innsbruck, Austria; 4 Department of General, Visceral and Vascular Surgery, Salzkammergut Klinikum, Vöcklabruck, Austria; Texas A&M University, UNITED STATES

## Abstract

**Background:**

To evaluate the efficacy, safety and overall clinical outcome of local treatment for recurrent intrahepatic cholangiocellular carcinoma after hepatic resection.

**Methods:**

Between 2007 and 2019 72 consecutive patients underwent hepatic resection for primary intrahepatic cholangiocellular carcinoma. If amenable, recurrent tumors were aggressively treated by HR or stereotactic radiofrequency ablation with local curative intent. Endpoints consisted **of** morbidity and mortality, locoregional and de novo recurrence, disease free survival, and overall survival.

**Results:**

After a median follow-up of 28 months, recurrence of intrahepatic cholangiocellular carcinoma was observed in 43 of 72 patients undergoing hepatic resection (60.3%). 16 patients were subsequently treated by hepatic resection (n = 5) and stereotactic radiofrequency ablation (n = 11) with local curative intention. The remaining 27 patients underwent palliative treatment for first recurrence. Overall survival of patients who underwent repeated aggressive liver-directed therapy was comparable to patients without recurrence (p = 0.938) and was better as compared to patients receiving palliative treatment (p = 0.018). The 5-year overall survival rates for patients without recurrence, the repeated liver-directed treatment group and the palliative treatment group were 54.3%, 47.7% and 12.3%, respectively. By adding stereotactic radiofrequency ablation as an alternative treatment option, the rate of curative re-treatment increased from 11.9% to 37.2%.

**Conclusion:**

Repeated hepatic resection is often precluded due to patient morbidity or anatomical and functional limitations. Due to the application of stereotactic radiofrequency ablation in case of recurrent intrahepatic cholangiocellular carcinoma, the number of patients treated with curative intent can be increased. This leads to favorable clinical outcome as compared to palliative treatment of intrahepatic cholangiocellular carcinoma recurrence.

## Introduction

Intrahepatic cholangiocarcinoma (ICC) is a rare but frequently fatal form of liver cancer. It is the second most common primary hepatic malignancy and its incidence and mortality rates have increased gradually in the past 30 years [1]. The long-term prognosis for ICC patients after curative hepatic resection (HR) remains poor, with a 5-year survival rate of 25–30% [2–5]. HR is the mainstay for potentially curative therapy in patients with resectable disease, but two-thirds of ICC patients who undergo resection will have recurrence, most often in the remnant liver [2, 6, 7]. Tumor recurrence occurs in 50–70% of cases at a median of 18 months and represents the limiting factor for long-term survival [5, 8–11]. Management of patients with recurrent disease is clinically challenging. Several researchers have examined the benefits of repeated local or systemic treatment for recurrent ICC, but there is still no consensus in the literature that define treatment for recurrent disease. Repeated resection for recurrent ICC has been reported as safe and technically feasible in selected cases and high-volume centres [8, 12, 13]. However, even with liver limited disease many patients cannot be considered as candidates for repeated resection. Either there are technical contraindications for HR, such as intolerable size and function of the future liver remnant (FLR), vascular structures at risk or an insufficient state of health of the patient precluding them from HR. Accordingly, several non-operative treatment options for ICC have been developed in order to improve the long-term outcomes for patients who are unfit for surgery [14].

In the recent past, conventional US- or CT-guided percutaneous radiofrequency ablation (RFA) techniques have been increasingly applied for the treatment of mainly unresectable primary and secondary liver tumours [15–23].

Reported 5-year overall survival (OS) rates after RFA of unresectable or recurrent ICC range from 15% to 30% [24–27].

3D-navigated ablation techniques such as stereotactic radiofrequency ablation (SRFA) rely on Karthesian coordinate systems. They allow for planning of multiple needle trajectories on 3D-CT/MRI and PET datasets. Three-dimensional navigation systems in combination with a rigid aiming device allow for precise probe placement according to the plan. Multiple overlapping ablation zones can be achieved which cover the complete tumour including a safety margin of 0.5 to 1 cm. This allows for local curative treatment of multiple and very large primary and secondary liver tumours [20, 28, 29].

Both local curative treatments, repeated HR and SRFA, could be beneficial in patients with recurrent ICC, but the efficacy of this strategy remains unclear. The aim of the present study was to compare the outcome of repeat curative intent liver-directed therapies with palliative systemic treatment for patients experiencing recurrent ICC after previous HR.

## Materials and methods

### Patient cohort

The clinical records of all consecutive patients undergoing HR with curative intent for ICC between 2007 and 2019 at Department of Visceral, Transplantation, and Thoracic Surgery at Medical University of Innsbruck were reviewed from a prospectively maintained database. Written informed consent for this retrospective analysis was obtained from all patients and all treatment decisions were obtained in multidisciplinary tumour board meetings. The protocol was approved by the Ethical Review Board committee of the Medical University Innsbruck (EC number 1076/2017 & AN4357 300/4.17). For index HR, all patients received primary surgery. Preoperative chemotherapy was an exclusion criterion.

### Operative procedures

Major hepatectomy was defined as a resection of 3 or more segments. Resections were performed either anatomically, non-anatomically or as a combination. Anatomical resections were classified according to the Brisbane 2000 Terminology [30]. In case of major hepatectomy assessment of liver function and calculation of the future liver remnant volume (FLRV) was performed on CT scans. As previously recommended, for patients with no underlying liver disease a FLRV of at least 20%, with steatosis 30%, and with cirrhosis (Child A) 40% was necessary [31, 32].

### Multi-probe Stereotactic Radiofrequency Ablation (SRFA)

The method of SRFA has been previously described in detail [33, 34]. In brief, the whole procedure is performed in an interventional CT room. The intubated patient is immobilized on the CT table by a vacuum mattress. 10–15 fiducials (X-SPOT, Beekley Corporation, Bristol, USA) are attached to the skin of the upper abdomen and a contrast-enhanced CT in the arterial and portal venous phase is acquired. To enable reproducible stereotactic conditions, the endotracheal tube is temporarily disconnected during the planning CT, each needle advancement and the final control CT.

Planning of the needle trajectories and overlapping ablation zones is performed on multiplanar reconstructions of the three-dimensional CT dataset with the software of the frameless stereotactic navigation system (Stealth Station Treon plus, Medtronic Inc., Louisville, USA). After registration using the skin markers, 15G x 17.2 cm coaxial needles (Bard Inc., Covington, USA) are sequentially advanced through the rigid targeting device to the pre-planned depth, as calculated by the planning software. For verification of coaxial needle placement, a native control CT with the needles in place is superimposed to the planning CT by using image fusion software of the navigation system. Biopsies may be obtained through the coaxial needles. Thereafter, three 17G RF-probes with 3cm tip exposures (Cool-tip, Medtronic, Mansfield, USA,) are introduced through the coaxial needles for serial tumour ablation. RFA is carried out with a unipolar ablation device including a switching controller (Cool-tip, Medtronic, Mansfield, USA). After ablation during probe withdrawal a contrast-enhanced CT in arterial and portal venous phase is obtained. The respective three-dimensional datasets are superimposed to the planning CT in order to verify complete coverage of the tumour by the ablation zone.

### Morbidity and mortality

In general, 90-day morbidity was defined as adverse events resulting in deviation from the normal postoperative course within 90 days after treatment. The severity of complications was assessed using the Clavien-Dindo classification, graded 1 to 5 [35]. Major complications were defined as grade 3a or higher, thereby including all patients requiring endoscopic, radiologic or surgical intervention. 90-day mortality was defined as death within 90 days after treatment.

### Treatment outcome

All patients operated before 2016 were regularly followed up for recurrence by clinical assessment and tumour markers every 3 months and CT every 6 months (ESMO guidelines 2009 [36]). For patients operated after 2016, follow-up consisted of 3-monthly visits during the first 2 years after therapy including clinical examination, laboratory investigation, tumour markers and CT scan, according to the ESMO guidelines 2016. After 2 years regular visits were extended to 6-monthly thereafter und prolonged to yearly visits after 5 years of follow-up [37]. Further work up with MRT was performed additionally if required. Surgical and oncological outcomes were evaluated based on our prospectively maintained database and patients’ clinical data files; the last date of follow-up included was the 18th of December 2020. Recurrence was defined as a lesion that was biopsy-proven recurrent ICC or a lesion that was deemed suspicious on cross-sectional imaging in the setting of an elevated CA19-9 level. OS data were cross-checked with the official, national registry on mortality maintained by Statistics Austria [38].

### Statistical analysis

Nominal variables are reported as frequencies and percentages and continuous variables as means or medians. Differences in continuous variables were analysed by Mann-Whitney U test, and differences in nominal variables were investigated by chi-square or Fisher’s exact test. The probability for DFS, recurrence-free survival (RFS), survival after recurrence (SAR) and OS was estimated by the Kaplan-Meier method and the results were compared by log-rank tests. DFS was calculated from the day of HR to date of diagnosis of first recurrence. RFS was measured from the day of first recurrence until the second recurrence. SAR was calculated from the day of first recurrence after index HR to the day of last follow-up or day of death. OS was assessed from the date of index HR to the date of death or the date of last follow-up. Two-sided p-values < 0.05 were considered as significant. Data analysis was performed using SPSS 24.0 (IBM Inc., USA).

## Results

### Patient and operative characteristics

**Fig 1:**
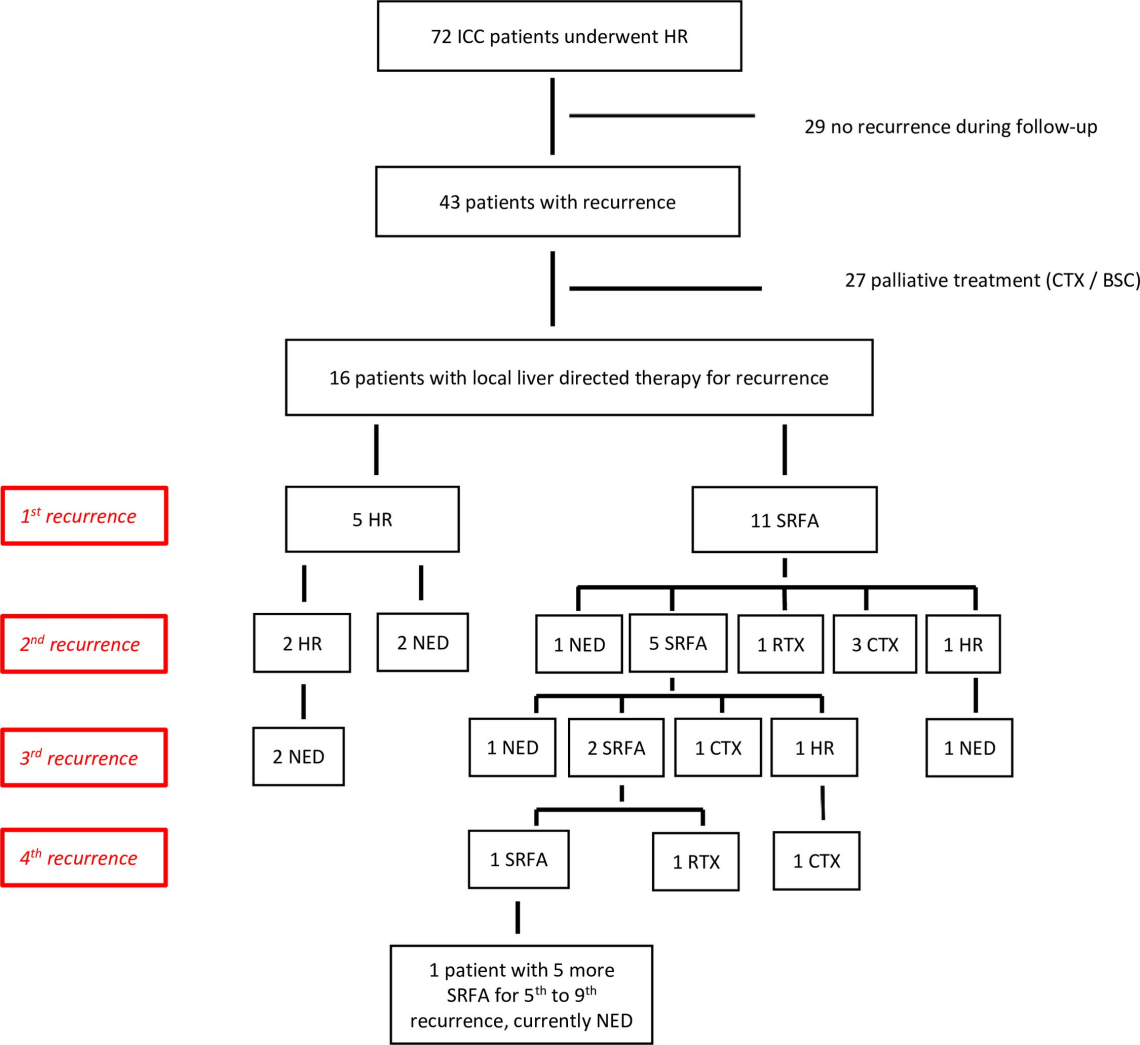
Flow diagram of the final cohort; ICC intrahepatic cholangiocarcinoma; HR hepatic resection; SRFA stereotactic radiofrequency ablation; CTX chemotherapy; RTX radiotherapy; BSC best supportive care.

A total of 72 patients who underwent HR with curative intent for histologically proven ICC were included in the final cohort (Fig 1). Demographic and clinical variables are summarized in Table 1.

**Table 1:** Demographic and clinical characteristics of 72 patients undergoing hepatic resection as first treatment.

Patient characteristics	Study group (n = 72)	
Female sex, n (%)	35	49.3
Age, median (range)	63	29–80
Cardiac comorbidity, n (%)	19	26.4
Pulmonary comorbidity, n (%)	6	8.3
Diabetes, n (%)	15	20.8
Cirrhosis, n (%)	23	31.9
Chronic kidney disease, n (%)	4	5.6
Tumour stage, n (%)		
T1	29	41.1
T2	26	37.1
T3	9	12.9
T4	6	8.6
Lymph node involvement	25	34.7
Bilobar disease, n (%)	19	26.4
Diameter of largest lesion, mm, median (range)	60	16–200
Number of lesions, median (range)	1	1–4
Major hepatectomy, n (%)	61	87.1
Type of resection, n (%)		
Anatomical	56	80.0
Non-anatomical	6	8.6
Combined resection	8	11.4
Type of anatomical resection, n (%)		
Right hepatectomy	13	19.7
Left hepatectomy	12	18.2
Extended right hepatectomy	22	33.3
Extended left hepatectomy	12	18.2
Left lateral sectorectomy	4	6.1
Segmentectomy	1	1.5
Bisegmentectomy	1	1.5
ALPPS	1	1.5
Laparoscopic approach, n (%)	5	7.1
R0, n (%)	64	88.9

mm millimetre, ALPPS Associating Liver Partition with Portal vein ligation for Staged hepatectomy

### Short-term outcome (< 90 days)

The median length of stay after HR was 16 days (4–102). Postoperative outcome in detail is shown in Table 2.

**Table 2:** Short-term outcome (<90days) following hepatic resection.

	Study group (n = 72)	
90-day morbidity, n %	34	47.2
90-day mortality, n%	2	2.8
Severe morbidity (CD≥3), n%	29	40.3
Haemorrhage, n %	5	6.9
Bile leakage, n %	13	18.1
POLF, n %	7	9.9
Grade B	1	1.4
Grade C	1	1.4
Reintervention, n %	13	18.1

CD Clavien Dindo classification; Bile leakage according to ISGLS classification[39]; POLF postoperative liver failure, Grade B & C according to ISGLS classification[40]

### Recurrence patterns and disease-free survival

Within a median follow-up of 28 months (3–174), 43 patients (60.3%) experienced tumour recurrence following index HR. Among the 43 patients with recurrence, 20 (46.5%) had liver limited recurrence (LLR), 6 (14.0%) experienced extrahepatic disease (EHD) and the remaining 17 (39.5%) patients developed both, LLR and EHD. Intrahepatic recurrence was located at the resection margin in 8 patients (21.5%), in 24 patients (65.0%) distant from the resection site (de novo tumour) and in 5 patients (13.5%) distant and local recurrence were noticed.

The median DFS after HR was 11 months (95%CI 7.1–14.2), with median 1-, 3- and 5-year DFS rates of 43.8%, 20.9% and 16.7%, respectively. The median DFS was independent from the pattern of recurrence (LLR 13 months, EHD 12 months, LLR&EHD 7 months, p = 0.321).

### Treatment for recurrence

Following first recurrence, local treatment with curative intent, namely HR and/or SRFA (in the following named as “curative intent”), could be applied in 16 patients. Treatment decisions were made in the interdisciplinary tumour board. If HR or SRFA were technically possible, treatment was performed according to patients’ preference or according to their physical condition. Repeated HR was performed in 5 patients, whereas 11 patients underwent SRFA. Of the 5 patients receiving repeated HR, SRFA was not possible in 2 cases due to location next to the central biliary tract. Among the 11 patients treated with SRFA, repeated HR was not an option due to an inadequate future liver remnant volume (FLRV, n = 5), a lymph node metastasis dorsal the inferior caval vein (n = 1) and patients’ condition/ comorbidity (n = 4). In one patient, SRFA with palliative intent was performed due to an initially suspicious pulmonary lesion, which was finally ruled out by histopathologic examination. Therefore, out of 43 patients with first recurrence, 26% were able to undergo another curative treatment even if surgery was not possible due to various reasons named above. 27 patients underwent palliative chemotherapy (n = 25) or best supportive care (n = 2) in the following named as “palliative intent”) due to diffuse multifocal intrahepatic and/or extrahepatic spread in 23 patients or centrally located lesions in 4 patients which could not be treated by HR or SRFA technically.

**Fig 2:**
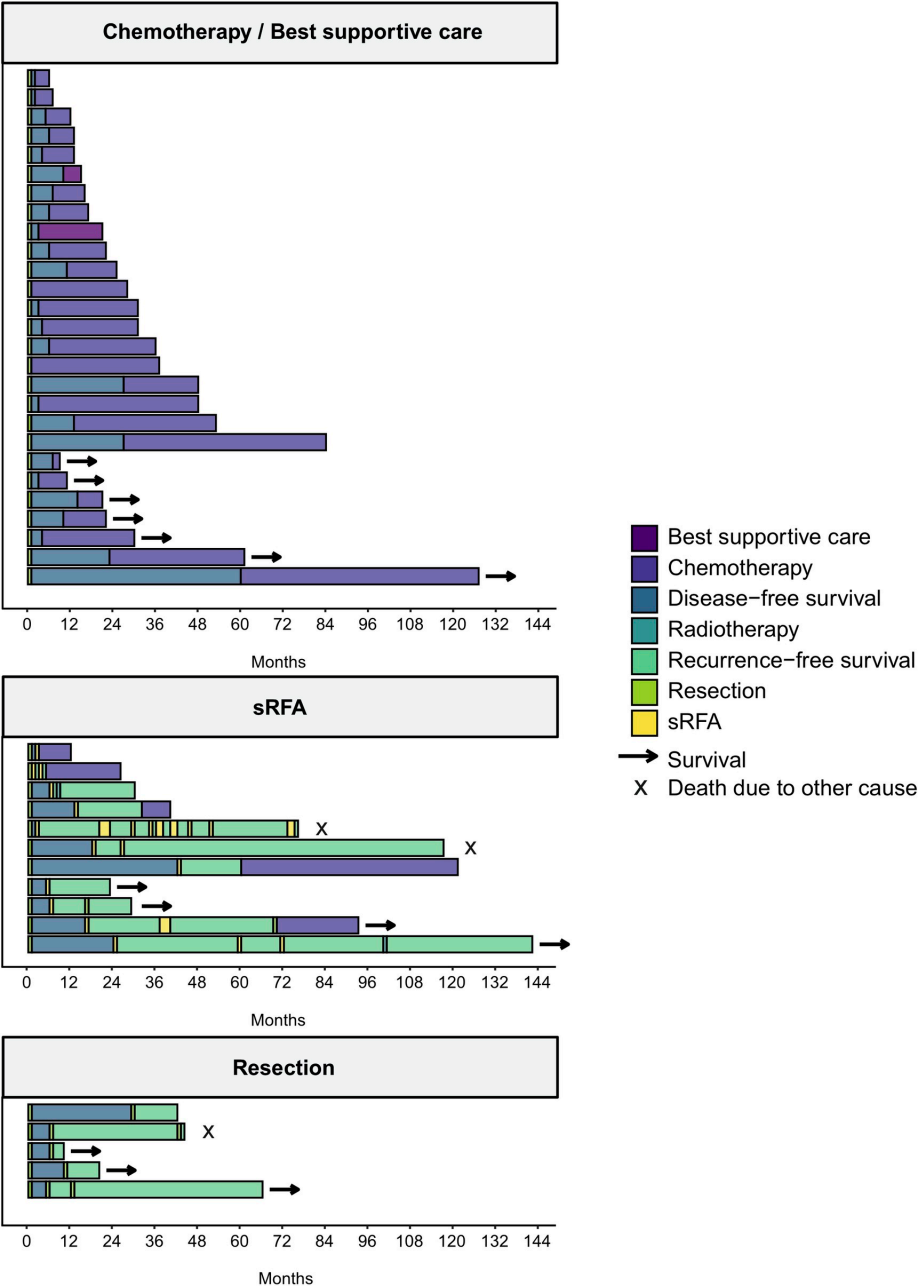
Treatment and survival of patients with recurrence following hepatic resection stratified by treatment for first recurrence; sRFA stereotactic radiofrequency ablation.

In 4/5 patients that were treated by HR for their recurrent ICC an R0 resection was achieved. 10/11 patients that were treated by SRFA did not show evidence of local recurrence in post-procedural CT scans. During follow-up, 12/16 patients developed second tumour recurrence. The RFS was comparable for both treatment types, 6 months (95% CI not calculable) for HR and for SRFA 8 months (95% CI 6.5–9.5), respectively (p = 0.269). Subsequent to the second recurrence, three patients received up to nine additional liver directed retreatments with curative intent during the course of their disease. A detailed diagram of all recurrent treatments during the course of the patient’s diseases is shown in Fig 2.

Of those, 12 patients (44.4%) received adjuvant chemotherapy. Keeping in mind that the BILCAP trial [41], published in 2019, demonstrated the first beneficial effect of adjuvant treatment and most of the patients in this collective were treated before 2019. Adjuvant chemotherapy consisted of Cisplatin/Gemcitabine (n = 6), Capecitabine (n = 3) and Gemcitabine (n = 3).

For palliative chemotherapy (n = 25), treatment also varied because no randomized, phase 3 data was available until the data from the ABC-02 trial which was published in 2010 [3]. In our study patients were mainly treated with Cisplatin/Gemcitabine (n = 10), Capecitabine (n = 3), Oxaliplatin (n = 3), FOLFOX (n = 3), Gemcitabine (n = 2), Capecitabine/ Oxaliplatin (n = 2), Gemcitabine/Oxaliplatin / Capecitabine (n = 1) and Gemcitabine/ Oxaliplatin (n = 1). Two patients received best supportive care due to their own request.

### Survival after recurrence

**Fig 3:**
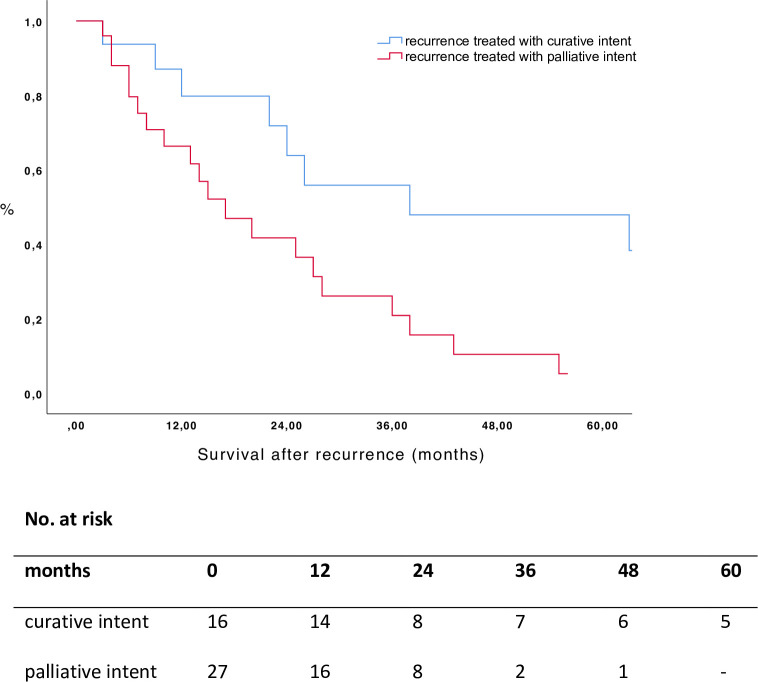
Survival after recurrence according to treatment group (p = 0.010, log rank test).

The median follow-up time after recurrence was 16 months (1–116). Overall median SAR was 24 months (1–117) and the 1-, 3- and 5-year survival rate were 73.4%, 32.4% and 22.7%, respectively. Patients who underwent curative intent treatments for their recurrences showed a better SAR compared to the group treated with palliative intent (p = 0.010, Fig 3, Table 3).

**Table 3:** Survival rates.

Survival		Median (months)	1-year survival rate (%)	3-year survival rate (%)	5-year survival rate (%)
DFS		11	43.8	20.9	16.7
SAR	curative intent	38	87.5	56.7	48.6
	palliative intent	17	64.8	16.6	0.0
OS	no recurrence	109	87.9	54.3	54.3
	curative intent	44	93.8	71.6	47.7
	palliative intent	31	88.6	36.0	12.3

DFS disease-free survival; SAR survival after recurrence; OS overall survival

### Overall survival

**Fig 4:**
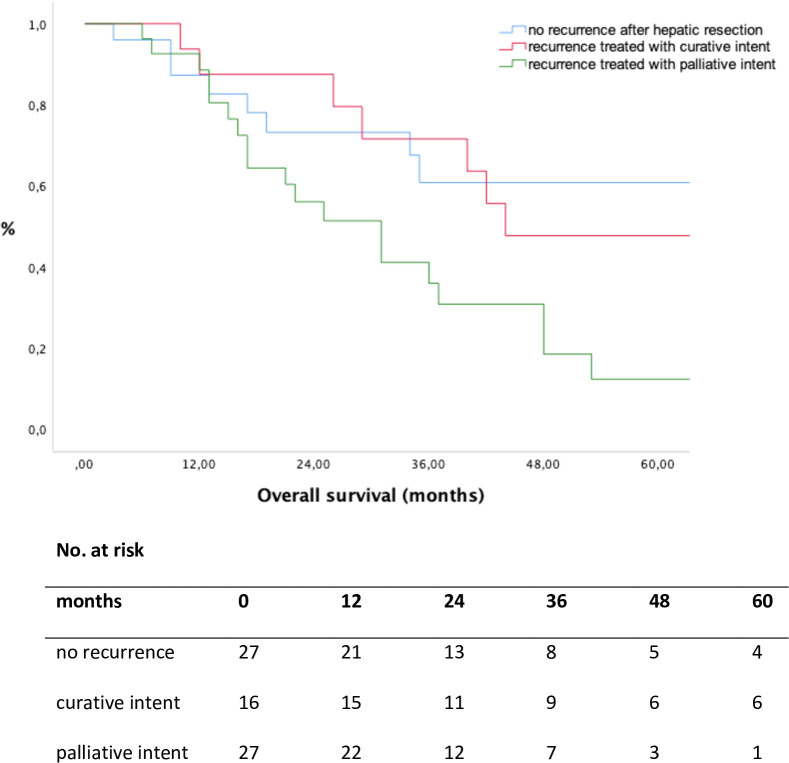
Overall survival according to treatment for recurrence, calculated from the day of initial hepatic resection. No recurrence vs. recurrence treated with curative intent (p = 0.938, log rank test); recurrence treated with curative intent vs. palliative intent (p = 0.018, log rank test).

Median OS of patients without recurrence after index HR was 109 months (1–174), 44 months (10–142) in patients treated with curative intent for recurrence and 31 months (6–84) in patients with palliative intent treatment of recurrence, respectively (Table 3). Comparing patients with no recurrence after HR with patients treated with curative intent, comparable OS rates could be shown (p = 0.938, Fig 4).

**Fig 5:**
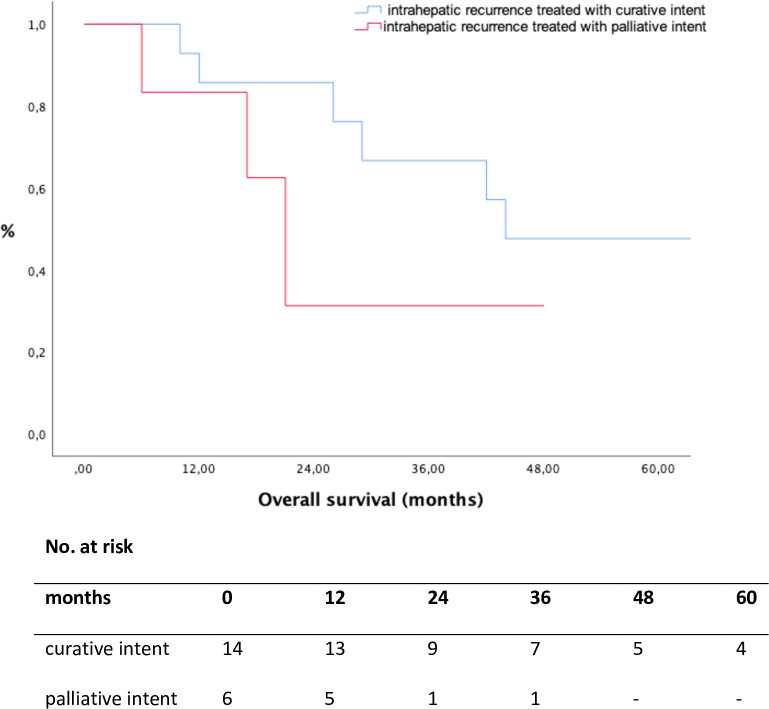
Overall survival according to treatment for recurrence in patients with liver limited recurrence (p = 0.027, log rank test).

In contrast, patients who underwent treatment with curative intent had superior OS rates compared to patients receiving palliative treatment (p = 0.018, Fig 4). The 5-year OS rates for patients without recurrence, the curative-intent group and the palliative intent group were 54.3%, 47.7% and 12.3%, respectively (Table 3). Considering all patients with LLR, patients treated with curative intent had a favorable OS compared to patients treated with palliative intent (p = 0.014, Fig 5).

## Discussion

ICC is a rare, life threatening malignancy with poor prognosis owing to a high rate of recurrence [4, 42]. In fact, data from the present study demonstrated that, despite a high rate of R0 resections (88.9%), locoregional recurrence and de novo lesions for ICC after HR are common. In this study, the incidence of recurrence after index HR was 60.3%, which is comparable to the literature [8, 10, 11, 13, 43]. Definitive guidelines for recurrent ICC are not available. Managing of recurrence for ICC has drawn increasing attention in the last decade. Some recent studies with even larger numbers of patients help to better delineate the role of repeated liver directed treatment for recurrent ICC [12, 44–47]. Although HR for recurrence is deemed to be the only curative treatment option, only selected patients with recurrence were eligible candidates for repeated resection in our study. Reasons to refrain from HR are a critical health state, anatomical limitations or an inadequate FLRV. Therefore, the aim of this study was to analyse survival outcomes of patients with recurrent ICC by applying different liver-directed therapies with local curative intent, namely SRFA and HR. In our series 43/72 patients developed recurrence. Of 37 patients with intrahepatic recurrence, 16 patients could be treated with curative intent. In our collective, of the 43 patients with recurrence, repeated HR was an option in only 5 patients (12%) whereas 11 patients (26%) could be treated by SRFA increasing the percentage of patients receiving treatment with local curative intent (according to their eligibility) from 12% to 38%. Increasing the number of patients receiving local treatment with curative intent for ICC recurrence represents the main goal of the application of SRFA in addition to HR in case of recurrent ICC. If repeated resection is considered the only curative option, other researchers reported a lower percentage of patients treated with curative intent for recurrence. For example, Sulpice et al. reported a re-resection rate of 16.0% [9], Souche et al. noted 14.0% [46] and Bartsch et al. performed a repeated HR in 16.8% [48].

RFA represents another option for the treatment of limited recurrent ICC [12, 24, 27]. SRFA is a sophisticated advancement of RFA, which allows local curative treatment of large and multiple tumours in one session by the achievement of overlapping ablation zones [49]. The success of this special method in various tumour entities has been described previously [22, 49–51]. Therefore, concerning the tumour size, SRFA seems to be a comparable option to HR which again leads to more therapeutical options. Due to the parenchyma sparing technique of percutaneous ablation, SRFA can be repeated multiple times in the same patient. During the course of his disease one patient underwent a total of 9 SRFA procedures after initial HR and is currently free of disease (after 75 months).

Besides the increasing number of patients receiving curative treatment for ICC, repeated liver-directed therapy with curative intent seems to be associated with longer OS compared to patients who received palliative treatment only (5-year OS rate 47.7% vs. 12.3%, Fig 4). Furthermore, patients who underwent repeated treatment with curative intent revealed comparable OS rates with patients without recurrence (5-year OS rate 54.3% vs. 47.7%, Fig 4). Despite complete cure, 4 patients died not because of tumour progression but from surgical complications, such as therapy-refractory liver abscess in 2 cases, one patient developed a necrosis of the main bile duct and one patient suffered from POLF grade C. These results may indicate a strong biological selection bias. However, even in patients with LLR, patients treated with curative intent for recurrent ICC had a favorable OS as compared to the “palliative intent” group (5-year OS rate 47.6% vs. 0%, Fig 5). The reason for these suitable results might be multifactorial, but the most plausible explanation is we are serving two different liver-directed treatment options with curative intent and using them according to the eligibility of the patient.

Hu et al. reported a better median OS in patients who underwent repeated curative-intent surgery compared to patients receiving palliative treatments (48.6 vs. 9.7 months). The 5-year OS rate in this study was 11.4% in patients who underwent repeated surgery and 4.1% in patients receiving systemic treatment [52]. Si et al. found that patients undergoing repeated HR achieved a median OS of 45.1 months with a 5-year OS rate of 41.9% [47]. Zhang et al. showed comparable 5-year OS rates (calculated from the day of diagnosis) in patients with repeated treatment (repeated HR in 81 cases, RFA in 22 cases, 63.7%) compared to those without ICC recurrence (77.1%) [53].

In a multicentre study by Spolverato et al., patients who underwent repeated hepatectomy reached longer OS than those treated with intra-arterial therapy or systemic chemotherapy (26.1 vs. 9.6 vs. 16.8 months) [8]. Randomized controlled trials comparing same tumour stage and patients`condition (ASA score, ECOG) would be desirable, but are still missing.

Given the high incidence of recurrent disease, understanding the patterns of recurrent ICC are crucial. In this study, nearly half of the patients developed recurrence within one year, with a median DFS of 11 months after index HR. These findings are comparable to the literature, with most recurrences occurring during the first two years after initial treatment [4, 6, 8–11, 13, 54, 55]. Isolated intrahepatic recurrence has been reported to be among the most common sites of recurrence for ICC after HR [4, 8, 53]. In fact, in the current study, LLR was seen in 46.5% of cases, EHD in 14.0% and LLR & EHD in 39.5%. Unlike most previous studies, we also identified differences in intrahepatic recurrence. Two thirds of patients with LLD (69.8%) developed distant spread, which corresponds a de novo tumour and 30.2% showed locoregional recurrence.

In our cohort, median DFS was independent from the pattern of recurrence (all patients 11 months, LLD 13 months vs. EHD 12 months). Spolverato et al. also found comparable 5-year DFS rates among patients who presented either LLD or EHD as the first site of their recurrence [8]. In contrast, Hu et al. reported that specific recurrence patterns had a different DFS. Patients who had a distant intrahepatic recurrence had a better DFS than patients with other patterns of recurrence [52]. Further studies are required to shed light on this issue.

Because of its small size, the study was not able to identify any morphologic or pathologic factors which helps in selecting patients for repeated treatment. This could be addressed by larger multicentre studies. However, aggressive liver-directed treatment may achieve favourable efficacy in treating intrahepatic recurrence under careful patient selection criteria and should be performed whenever possible. Unfortunately, a set of patients with recurrent disease will not be candidate for repeated HR or SRFA. These patients may benefit from systemic chemotherapy [3, 56, 57].

The present study contains several limitations. The main limitation is the selection bias. Prospective, randomized controlled trials on patients treated with chemotherapy vs. chemotherapy in combination with HR/ SRFA would be desirable to diminish this bias. As in other similar studies, the small sample size is rather low. Therefore, the conclusion regarding the role of liver-directed therapy has to be taken with caution. However, an increasing number of low sample size studies report analogous results indicating that aggressive liver-directed therapy with local curative intent is a favourable option in recurrent ICC. A further limitation of this study is the retrospective design, which resulted in varied durations of follow-up and loss to follow-up. Nevertheless, survival status was updated before starting the analysis. Well designed, larger, multicentre studies are desirable to draw final conclusions.

## Conclusion

In conclusion, repeated local liver-directed curative treatment, when feasible, is associated with prolonged OS, as compared to systemic treatment alone. Besides repeated resection SRFA represents a viable and attractive option to achieve this goal. Due to the application of SRFA for recurrent ICC the number of patients receiving treatment with curative intent can be significantly increased.

## Supporting information

S1 Dataset (XLSX)
